# *QuickStats:* Percentage* of Adults Aged 18–64 Years Who Had Seen or Talked to a Health Care Professional in the Past 12 Months,^†^ by Race/Ethnicity^§^ — National Health Interview Survey, 2012–2013 and 2017–2018^¶^

**DOI:** 10.15585/mmwr.mm6848a3

**Published:** 2019-12-06

**Figure:**
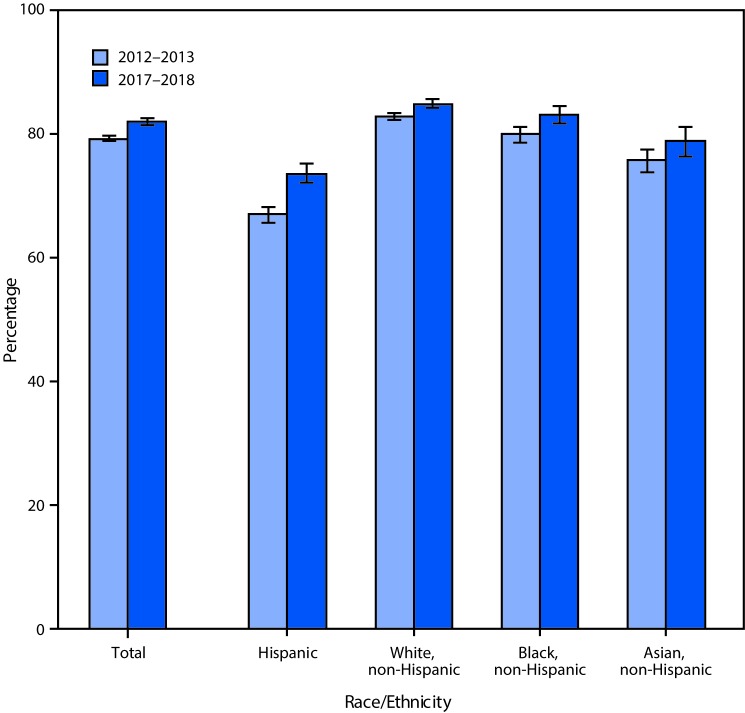
The percentage of adults aged 18–64 years who had seen or talked to a health care professional in the past 12 months increased from 79.3% in 2012–2013 to 82.1% in 2017–2018. There was an increase in the percentage of Hispanic (67.0% to 73.6%), non-Hispanic white (82.8% to 84.9%), non-Hispanic black (80.0% to 83.2%), and non-Hispanic Asian (75.8% to 78.8%) adults who had seen or talked to a health care professional in the past 12 months between those two periods. During 2012–2013 as well as 2017–2018, non-Hispanic white adults were the most likely and Hispanic adults were the least likely to have seen or talked to a health care professional in the past 12 months.

**Source:** National Health Interview Survey, 2012, 2013, 2017, and 2018 data. https://www.cdc.gov/nchs/nhis.htm.

